# Survival Prediction in Patients with Hypertensive Chronic Kidney Disease in Intensive Care Unit: A Retrospective Analysis Based on the MIMIC-III Database

**DOI:** 10.1155/2022/3377030

**Published:** 2022-05-12

**Authors:** Zuoxun Xia, Peng Xu, Ye Xiong, Yunbo Lai, Zhaohui Huang

**Affiliations:** ^1^Guizhou Medical University, China; ^2^The Department of Infectious Diseases, State Key Laboratory for Diagnosis and Treatment of Infectious Diseases, National Clinical Research Center for Infectious Diseases, Collaborative Innovation Center for Diagnosis and Treatment of Infectious Diseases, The First Affiliated Hospital, Zhejiang University School of Medicine, China; ^3^Guiqian International General Hospital, China; ^4^Department of Nephrology and Rheumatology, The Affiliated Hospital of Guizhou Medical University, Guiyang, Guizhou, China

## Abstract

**Objective:**

Disease prediction is crucial to treatment success. The aim of this study was to accurately and explicably predict, based on the first laboratory measurements, medications, and demographic information, the risk of death in patients with hypertensive chronic kidney disease within 1 and 3 years after admission to the Intensive Care Unit (ICU).

**Methods:**

Patients with hypertensive chronic kidney disease who had been registered in the Medical Information Mart for Intensive Care (MIMIC-III) database of critical care medicine were set as the subject of study, which was randomly divided into a training set and a validation set in a ratio of 7 : 3. Univariate Cox regression analysis and stepwise Cox regression analysis were applied in the training set to identify the predictive factors of prognosis of patients with hypertensive chronic kidney disease in ICU, and the predictive nomogram based on Cox regression model was constructed. We internally validated the model in the training set and externally validated that in the validation model. The efficacy was assessed primarily through area under the receiver operating characteristic (ROC) curve, clinical decision curves, and calibration curves.

**Results:**

A total of 1762 patients with hypertensive chronic kidney disease were finally included. During the 3-year follow-up, 667 patients (37.85%) died, with a median follow-up time of 220 days (1-1090). The data set were randomly divided into a training set (*n* = 1231) and a validation set (*n* = 531). It was identified in the training set that insurance, albumin, alkaline phosphatase, the mean corpuscular hemoglobin concentration, mean corpuscular volume, history of coronary angiogram, hyperlipemia, medication of digoxin, acute renal failure, and history of renal surgery were the most relevant features. Taking 1 year and 3 years as the cut-off points, the AUC of participants were 0.736 and 0.744, respectively, in the internal validation and were 0.775 and 0.769, respectively, in the external validation, suggesting that the model is of favorable predictive efficacy.

**Conclusion:**

We trained and validated a model using data from a large multicenter cohort, which has considerable predictive performance on an individual scale and could be used to improve treatment strategies.

## 1. Introduction

Hypertension is closely related to chronic kidney diseases (CKD). Kidney is the primary target of hypertension-induced target organ injury. Hypertension is the main cause of end-stage renal disease. It has been demonstrated that CKD, including that caused by hypertension, is a robust independent risk factor of cardiovascular adverse events and significantly affects the prognosis of patients [1, 2]. Conventional complications induced by CKD (e.g., electrolyte disorder and uremia), which could be addressed via hemodialysis, account for only 3 percent of kidney injury-related death in ICU [3]. Several studies have shown that the high mortality of kidney injury is associated with multiple organ dysfunction in that it could increase the susceptibility to infection and the morbidity of respiratory failure and could compromise cardiac function directly and indirectly [4]. With regard to hypertension, it is correlated with multiple cardiac performances including left ventricular hypertrophy, congestive heart failure, arrhythmia, and ischemic heart disease [5]. To date, there are many studies focusing on the prognosis of various types of hypertension and CKD, while little attentions have been paid to the prognostic factors of patients with hypertensive chronic kidney disease [6–8]. It remains unclear that the prognostic factors of patients with hypertensive chronic kidney disease would be different due to the dual damage they suffer from both hypertension and CKD.

Nomogram is a pictorial representation of a mathematical formula. They are commonly used tools to estimate prognosis in medicine. With the ability to generate an individual numerical probability of a clinical event by integrating diverse prognostic and determinant variables, nomograms fulfill our drive towards personalized medicine. Therefore, nomogram is an available approach for the exploration of risk factors related to the prognosis of patients with hypertensive chronic kidney disease, which is visual, and can stratify the risk more accurately so as to provide information for clinical decision [9, 10]. It performs excellently in many research fields [11–13]. The earlier the correct clinical decisions are made, the better the patients would be benefited. Therefore, the data we used for training were the first measurements of patients after their admission to ICU, which made our predictions more prospective. Additionally, medications during the treatment may also result in adverse consequences for patients so that we included medications as variables to explore its potential effects on patients' prognosis. Our aim was to train and independently validate a nomographic model that could be applied to predict the prognosis of patients when they are admitted to ICU, so as to develop strategies that would be more beneficial.

## 2. Methods

### 2.1. Data Sources

The data of this study was from the MIMIC-III database of critical care medicine. The MIMIC-III is a comprehensive data set containing demographic information, laboratory measurements, diagnosis, medication, survival, and scores of related assessments of each participant during ICU admission, which could be used for prognostic analysis.

All patients registered in the MIMIC-III who have been diagnosed as hypertensive chronic kidney disease were included in this study, and the following variables with the missing data less than 20% were included:

1. Basic information: ethnicity, gender, time of survival, living situation, and insurance/marital status
2. Laboratory measurements: white blood cell count (WBC), creatinine, blood acid-based scale (pH), partial pressure of carbon dioxide (PCO^2^), partial pressure of oxygen (PO^2^), albumin (ALB), blood glucose, lactic dehydrogenase (LDH), serum magnesium ion concentration, MCH, MCHC, MCV, monocytes, neutrophil counts, platelet count, PT, red blood cell count (RBC), etc.
3. Concomitants diseases: acute renal failure, infection, diabetes, and hyperlipidemia
4. History of renal surgery
5. Medication: phenytoin, digoxin, vancomycin, and Tylenol

Variables referring to diseases and surgical history (e.g., hypertensive chronic kidney disease, acute renal failure, diabetes, hyperlipidemia, and renal surgery) were arranged in accordance with the 9^th^ revision of the International Classification of Diseases (ICD-9). All laboratory data were the first measurements after patients' admission to ICU. The data set was randomly divided into a data set for training and a data set for validation in a ratio of 7 : 3 prior to the analysis. The training set was assigned to modeling and internal validation while the validation set to external validation

### 2.2. Statistical Analyses

#### 2.2.1. Features Selection

We selected the features of patients (demographic information, the first laboratory measurements after ICU admission, medical history, surgical history, medications, etc.) from the data set. Two steps were taken to the full screening of all the features in order to evaluate the predictive potential of each feature (a *P* < 0.05 was deemed as statistically significant).

Step 1. Univariate Cox regression analysis was conducted using *Ezcox R (Version 1.0.2)* to identify correlations between the variables and the prognosis of patients and to remove features that are statistically insignificant

Step 2. Reserved variables after step 1 were included in stepwise Cox regression analysis for further screening to simplifying the model

#### 2.2.2. Predictions and Verifications

Cox regression model was performed using *Survival R (Version 3.2.13)* to predict the risk of death within 1 year and 3 years after ICU admission. After modeling, the training set and the validation set were included in the model to conduct the internal and external validation, respectively. Predicted values of the model were calculated. ROC curves, clinical decision curves, and calibration curves were plotted to verify the efficacy of the model. All statistical analyses were processed using R Version 4.1.2.

## 3. Results

### 3.1. Cohort Description

1762 participants with hypertensive chronic kidney disease were retained for the final analysis after removing patients with missing data or without ICU admission, of whom 1090 were male (61.86%) and 672 were female (38.14%). The overall survival time was 1110.19 ± 702.69 days, and the median survival time was 1460 days. There were 667 deaths (37.75%) during the first 3 years after diagnosis. The data set were divided into a training set and a validation set in a ratio of 7 : 3. There were 1231 patients in the training set with 765 male (62.14%) and 466 female (37.86%). The overall survival time in the training set was 1111.44 ± 702.16 days with the maximum of 3798 and the minimum of 1. There were 531 patients in the validation set with 325 male (61.21%) and 206 female 38.79%. The overall survival time was 1107.27 ± 704.57 days with the maximum of 3606 and the minimum of 1. The basic information of patients and variables are shown in Table 1.

#### 3.1.1. Features Selection

The univariate Cox regression suggested that insurance, marital status, ethnicity, ALB, alkaline phosphatase (ALP), MCHC, MCV, neutrophil, PT, RBC, history of coronary angiogram, acute renal failure, hyperlipemia, history of renal surgery, and medication history of digoxin and vancomycin are the independent influence factors for the prognosis of hypertensive chronic kidney disease. Variables with statistical significance in the univariate analysis were included in the stepwise Cox regression, and the results suggested that insurance, albumin, alkaline phosphatase, MCHC, MCV, history of coronary angiogram, hyperlipemia, medication history of digoxin, acute renal failure, and history of renal surgery had stronger independent effects on the prognosis of hypertensive chronic kidney disease (Table 2).

#### 3.1.2. Cox Model and Nomogram

**Figure 1:**
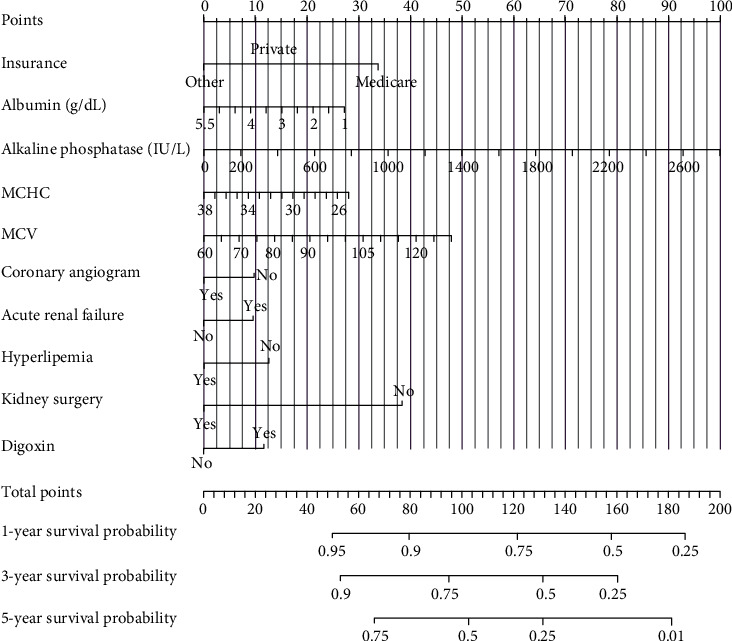
The nomogram of Cox regression model.

The selected features were included in the Cox regression model for modeling (Table 3), and the nomogram were plotted (Figure 1). The C-index of Cox model is 0.703. Factors in the final model for predicting CSD were insurance (private vs. Medicare: Coef = −0.5691; private vs. other: Coef = −0.8148), albumin (Coef = −0.1455, *P* = 0.0251), alkaline phosphatase (Coef = 0.0009, *P* = 0.0058), MCHC (Coef = −0.0520, *P* = 0.0468), MCV (Coef = 0.0165, *P* = 0.0011), coronary angiogram (yes vs. no: Coef = −0.2332, *P* = 0.0404), acute renal failure (yes vs. no: Coef = 0.2276, *P* = 0.0058), hyperlipemia (yes vs. no: Coef = −0.3016, *P* = 0.0003), digoxin (yes vs. no: Coef = 0.2771, *P* = 0.0294), kidney surgery (yes vs. no: Coef = −0.9228, *P* < 0.0001). The results showed that alkaline phosphatase, MCV, acute renal failure, and digoxin increased the mortality of patients with hypertensive chronic kidney disease, while insurance, albumin, MCHC, coronary angiogram, hyperlipemia, and kidney surgery decreased the mortality.

#### 3.1.3. Model Performances

**Figure 2:**
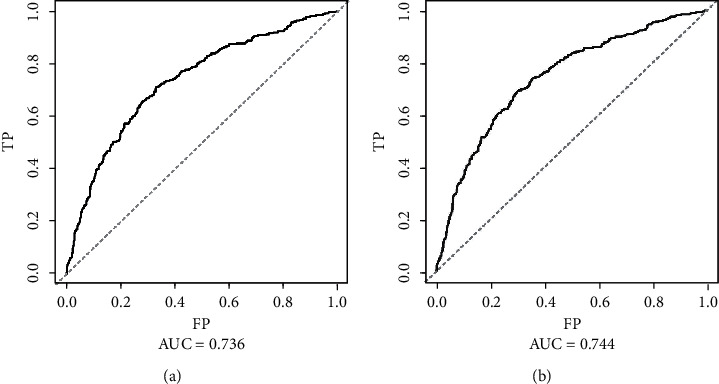
The ROC curves of internal validation: (a) The mortality that occurred within one year after admission to ICU; (b) the mortality that occurred within three years after admission to ICU.

**Figure 3:**
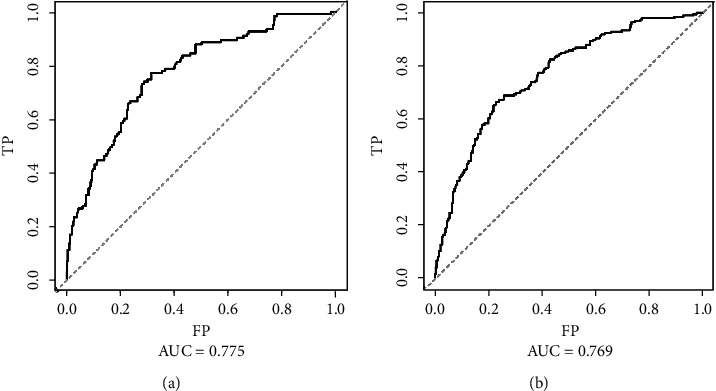
The ROC curves of external validation: (a) The mortality that occurred within one year after admission to ICU; (b) the mortality that occurred within three years after admission to ICU.

**Figure 4:**
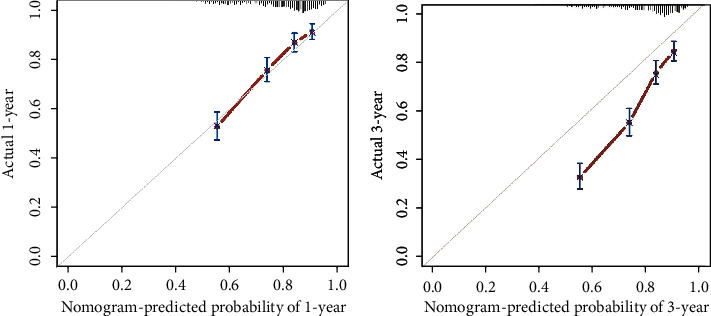
The calibration curves in the internal validation cohort.

**Figure 5:**
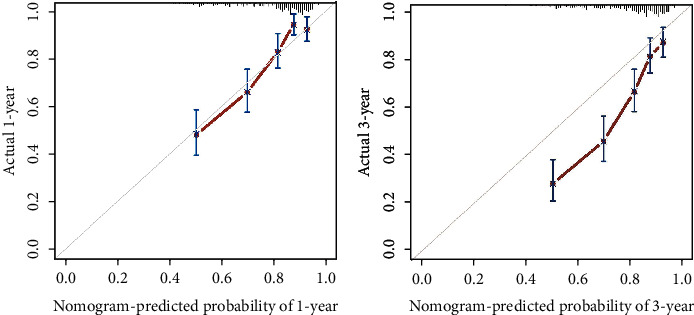
The calibration curves in the external validation cohort.

**Figure 6:**
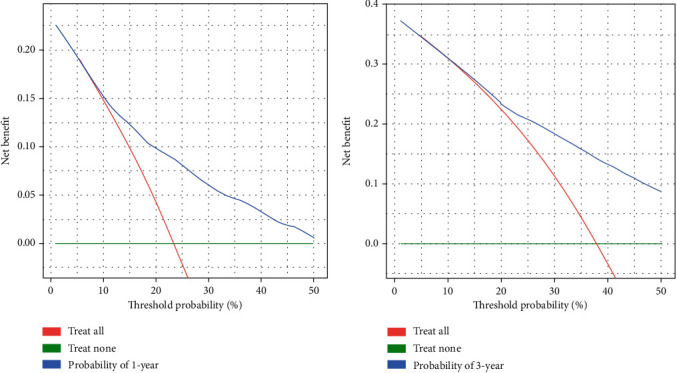
The decision curves in the internal validation cohort.

**Figure 7:**
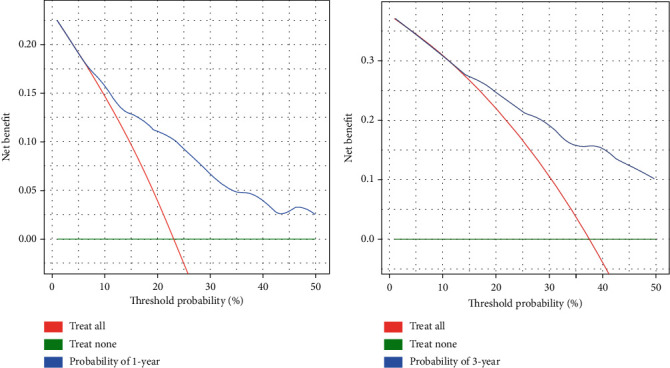
The decision curves in the external validation cohort.

In the internal validation, the area under the ROC curves (AUC) of one year and three years were 0.736 and 0.744, respectively. (Figure 2). In the external validation, the area under the ROC curves of one year and three years were 0.775 and 0.769, respectively (Figure 3). The AUC value indicated good prognostic prediction efficacy in both validation sets. In addition, the calibration curves for 1 year showed favorable calibration in the internal (Figure 4) and external (Figure 5) validation set and showed a good agreement between predicted and observed outcomes for the nomogram. To evaluate the clinical utility of the nomogram, decision curve analysis (DCA) was used (internal validation: Figure 6; external validation: Figure 7). The DCA curves showed good positive net benefits in the prognostic model among most of the threshold probabilities at different time points (death at 1 and 3 years).

## 4. Discussion

Our study revealed that the nomographic model based on MIMIC-III could make a comparatively accurate prediction for the prognosis of patients with hypertensive chronic kidney disease who were admitted to ICU (the AUC of 1 year and 3 years were, respectively, 0.775 and 0.769 in the internal validation and were, respectively, 0.775 and 0.769 in the external validation), suggesting its potent predictive efficacy.

However, there is something controversial. According to the nomographic model, concomitance of hyperlipemia and history of renal surgery could result in increased survival rates, which is the opposite of what we perceived. We presumed that these two variables might be confounded by a certain variable; thus, we plotted the nomogram through a stepwise removing of variables in the Cox model, and there is no reverse in the variable directions of concomitant hyperlipemia and history of renal surgery. This could rule out the possibility of potential effects on these two variables by others. Despite the fact that multiple studies indicated that hyperlipemia increases the risk of cardiovascular diseases and pancreatitis, it is chronic and there is no evidence to support that hyperlipemia might increase the risk of death in critical patients admitted to ICU [14]. A study discovered that increased levels of lipometabolism-associated fatty acids and ketones were physiologically promotive for nutrition utilization, and elevated blood lipids might play a positive role in critical patients to whom nutritional support is needed [15]. Moreover, an animal study by Miyamoto et al. illustrated that adipokines played a protective role in the pathogenesis of CKD through several pathways and might be potential in the prevention of CDK progression [16]. The intake of lipid emulsion could also lead to increased blood lipids during nutritional support for critical patients, which might interfere with the diagnosis of hyperlipemia [17]. With regard to history of renal surgery, we identified the surgery types of cases in the training set. Among the 1231 patients, 697 had undergone renal surgery, of which only 51 were more invasive (nephrotomy and nephrostomy, local excision or destruction of lesion or tissue of kidney, and complete nephrectomy), most of the rest are diagnostic procedures on the kidney. We could generally accept that renal surgery is of therapeutic benefits and is beneficial to the prognosis of patients.

On the other hand, we found that MCV had a significantly positive correlation with the risk of death of the study cohort (*P* = 0.0011). MCV is often used for diagnosis of anemia of various types in clinical practice, and most of the related studies were focusing on anemia [18–20]. However, a retrospective analysis of a CKD cohort (*n* = 1439) by Hsieh et al. reported that MCV was significantly and positively correlated with all-cause mortality, cardiovascular diseases-related mortality, and infection-related mortality and was independent of other factors, which was consistent with that observed in our study [21]. Our results also showed that history of coronary angiogram could increase the survival rate of patients (*P* = 0.0404), while the use of digoxin could increase the mortality (*P* = 0.0294), which was consistent with our expectation. These two features are cardiovascular diseases-related, and cardiovascular function is crucial to the prognosis of patients in ICU. First, patients in ICU are persistent bedridden and usually receive peripherally inserted central catheters (PICC). Being bedridden could induce deep venous thrombosis (DVT) [22], while PICC is associated with upper extremity venous thrombosis [23], and both could result in several serious events like postthrombotic syndrome and pulmonary embolism [24]. Hypercoagulable state of blood is a critical risk factor for thrombosis [25]. Secondary, hemodynamic abnormity could also lead to poor interaction between critical patients and ventilator in ICU [26, 27]. Therefore, the cardiovascular function and hemodynamics of critical patients should be far more concerned to timely avoid the potential risk.

Our results demonstrated that the nomographic model could provide extra value for the prediction of the prognosis of patients with hypertensive chronic kidney disease in ICU. The predicted variables are explainable and dependable for ICU clinicians and could improve the clinical utilization to save the costs of treatment and time. Another merit of our study is that the laboratory data included in the training model were all the first measurements after the patients were admitted to ICU, which suggests that clinical practitioners can make better treatment strategies in the light of our nomographic model as soon as patients are admitted to ICU. The nomogram, different from the machine learning model, is visualizable, handy, and generally applicable. Though the machine learning model, such as the neural network and the random forests, could reveal the complicated nonlinear relation between different features, it comes with a price known as the “Black-Box” effect making it difficult for the determination of weights of the features [28]. Our study has a limitation. Despite the diversity and comprehensiveness of data that the MIMIC-III contains, it is not specifically designed for our specific ends. We had to rule out variables with sample size missing to ensure enough samples for model training, even they were interesting. This limitation is expected to be lifted with the abundance of MIMIC database and the increasing sample size of data.

Though the limitation our study has, the nomographic model performed well and could optimize the clinical decision-making of clinicians in ICU. The nomogram, for instance, scored the overall unfavorable prognosis for patients with increased MCV level, then, whether clinicians should initiate the intensive treatment? According to the explanation of predicted features, they could decide to initiate or not to initiate the intensive treatment, to achieve a positive therapeutic effect.

## 5. Conclusion

The nomographic model presented in this study could predict the risk of death in patients at the beginning of their admission to ICU. It is highly perspective, explainable, reliable, and clinically stable. The results showed that elevated MCV and ALP, acute renal failure, and medication of digoxin could increase the risk of death in ICU-admitted patients with hypertensive chronic kidney disease, while elevated ALB and MCHC, history of renal surgery, concomitance of hyperlipemia, and history of coronary angiogram could decrease the risk of death in the patients. Among these, the conclusion that concomitance of hyperlipemia could reduce the risk of death in critical patients is still controversial and remains to be further discussed.

## Figures and Tables

**Table 1:** A description of all the variables.

	Alive (*n* = 835)	Dead (*n* = 927)	All (*n* = 1762)
Gender (male/female)	507/328	583/344	1090/672
Ethnicity (white/black/other)	570/178/87	710/153/64	1280/331/151
Married (yes/no)	434/401	449/478	883/879
Insurance (Medicare/private/other)	526/220/89	714/146/67	1240/366/156
Acute renal failure (no/yes)	405/430	461/466	866/896
Coronary angiogram (no/yes)	714/131	718/209	1432/330
Diabetes (no/yes)	338/497	399/528	737/1025
Hyperlipemia (no/yes)	382/453	567/360	949/813
Kidney surgery (no/yes)	203/632	552/375	755/1007
Blood (urine) (negative/small/moderate/large)	324/107/90/314	337/109/122/359	661/216/212/673
Bilirubin (negative/small/moderate/large)	745/68/10/12	800/103/14/10	1545/171/24/22
Dilantin (no/yes)	812/23	857/70	1669/93
Digoxin (no/yes)	810/25	827/100	1637/125
Vancomycin (no/yes)	667/168	512/415	1179/583
Tylenol (no/yes)	794/41	878/49	1672/90
WBC	10.35 ± 7.62	10.46 ± 5.62	10.41 ± 6.64
Creatinine	3.09 ± 3.21	3.04 ± 2.53	3.06 ± 2.87
PH	7.35 ± 0.11	7.35 ± 0.11	7.35 ± 0.11
PCO2	42.01 ± 12.48	42.33 ± 13.84	42.18 ± 13.21
PO2	167.69 ± 131.27	151.51 ± 117.72	159.17 ± 124.55
Albumin (g/dL)	3.41 ± 0.66	3.25 ± 0.61	3.33 ± 0.64
Alkaline phosphatase (IU/L)	113.85 ± 110.97	129.05 ± 143.53	121.85 ± 125.76
Anion gap (mEq/L)	17.11 ± 4.99	17.13 ± 4.37	17.12 ± 4.67
Bicarbonate (mEq/L)	23.85 ± 5.46	24.01 ± 5.33	23.93 ± 5.39
Bilirubin total (mg/dL)	1.03 ± 2.98	1.01 ± 2.17	1.02 ± 2.59
CK (IU/L)	416.80 ± 2154.17	258.09 ± 746.04	333.30 ± 1580.08
Glucose (mg/dL)	171.77 ± 128.92	166.08 ± 113.23	168.77 ± 120.92
LD (IU/L)	357.15 ± 633.63	344.52 ± 462.63	350.51 ± 550.20
Magnesium (mg/dL)	2.02 ± 0.44	2.02 ± 0.44	2.03 ± 0.43
MCH	29.88 ± 2.80	30.18 ± 2.84	30.03 ± 2.82
MCHC	33.21 ± 1.67	33.10 ± 1.52	33.15 ± 1.59
MCV	89.98 ± 7.16	91.22 ± 7.74	90.64 ± 7.50
Monocytes	4.79 ± 2.83	4.99 ± 3.45	4.89 ± 3.71
Neutrophils	75.65 ± 13.67	76.71 ± 13.16	76.21 ± 13.41
Platelet count (K/*μ*L)	247.30 ± 115.87	238.23 ± 115.31	242.53 ± 115.63
PT	16.08 ± 9.54	16.09 ± 7.79	16.08 ± 8.66
RBC	3.82 ± 0.73	3.77 ± 0.75	3.79 ± 0.74
Time	1460.00 ± 0.00	795.09 ± 853.98	1110.19 ± 702.69

**Table 2:** Features selection (univariate and multivariate analyses).

Variable		Univariate analysis		Multivariate analysis	
		HR (95% CI)	*P*	HR (95% CI)	*P*
Insurance	Medicare (ref)				
	Private	0.59 (0.48-0.73)	<0.0001	0.57 (0.46-0.70)	<0.0001
	Other	0.49 (0.36-0.68)	<0.0001	0.44 (0.32-0.61)	<0.0001
Married (no)		1.18 (1.01-1.37)	0.0373		
Ethnicity	White (ref)				
	Black	0.75 (0.60-0.93)	0.0080		
	Other	0.85 (0.63-1.14)	0.2740		
Gender (female)		0.87 (0.74-1.02)	0.0775		
WBC (K/*μ*L)		1.01 (1.00-1.02)	0.1420		
Creatinine		1.01 (0.98-1.03)	0.6770		
PCO2		1.00 (1.00-1.01)	0.7550		
PH		0.89 (0.45-1.74)	0.7230		
PO2		1.00 (1.00-1.00)	0.1680		
Albumin (g/dL)		0.79 (0.70-0.89)	<0.0001	0.86 (0.76-0.98)	0.0252
Alkaline phosphatase (IU/L)		1.00 (1.00-1.00)	0.0003	1.00 (1.00-1.00)	0.0059
Anion gap (mEq/L)		1.00 (0.98-1.01)	0.7790		
Bicarbonate (mEq/L)		0.99 (0.98-1.01)	0.2760		
Bilirubin Total (mg/dL)		1.01 (0.98-1.04)	0.4780		
CK (IU/L)		1.00 (1.00-1.00)	0.1500		
Glucose (mg/dL)		1.00 (1.00-1.00)	0.2580		
LD (IU/L)		1.00 (1.00-1.00)	0.4240		
Magnesium (mg/dL)		1.14 (0.96-1.36)	0.1330		
MCH (pg)		1.01 (0.99-1.04)	0.3020		
MCHC		0.92 (0.87-0.96)	0.0002	0.95 (0.9-1.00)	0.0471
MCV (f/L)		1.02 (1.01-1.03)	0.0008	1.02 (1.01-1.03)	0.0010
Monocytes		1.00 (0.97-1.02)	0.8530		
Neutrophils		1.01 (1.00-1.01)	0.0100		
Platelet count (K/*μ*L)		1.00 (1.00-1.00)	0.3570		
PT		1.01 (1.00-1.02)	0.0100		
RBC (m/*μ*L)		0.83 (0.75-0.92)	0.0002		
Coronary angiogram (yes)		0.69 (0.55-0.86)	0.0008	0.79 (0.63-0.99)	0.0406
Acute renal failure (yes)		1.31 (1.12-1.54)	0.0006	1.26 (1.07-1.48)	0.0058
Infection (yes)		1.19 (0.98-1.44)	0.0760		
Blood (urine)	Negative (ref)				
	Small	1.13 (0.87-1.47)	0.3480		
	Moderate	1.29 (1.00-1.65)	0.0510		
	Large	1.11 (0.93-1.33)	0.2350		
	Negative (ref)				
Bilirubin	Small	1.27 (0.99-1.62)	0.0574		
	Moderate	1.61 (0.80-3.24)	0.1820		
	Large	1.16 (0.60-2.25)	0.6550		
Kidney surgery (yes)		0.37 (0.32-0.43)	<0.0001	0.40 (0.34-0.47)	<0.0001
Diabetes (yes)		0.88 (0.76-1.03)	0.1170		
Hyperlipemia (yes)		0.58 (0.50-0.68)	<0.0001	0.74 (0.63-0.87)	0.0003
Dilantin (yes)		1.35 (0.99-1.85)	0.0600		
Digoxin (yes)		1.79 (1.40-2.29)	<0.0001	1.32 (1.03-1.69)	0.0029
Vancomycin (yes)		1.66 (1.42-1.94)	<0.0001		
Tylenol (yes)		0.81 (0.57-1.14)	0.2190		

**Table 3:** Cox regression model.

		Coefficients	S.E.	Wald	*P*
Insurance	Medicare (ref)				
	Private	-0.5691	0.1086	-5.24	<0.0001
	Other	-0.8148	0.1620	-5.03	<0.0001
Albumin (g/dL)		-0.1455	0.0650	-2.24	0.0251
Alkaline phosphatase (IU/L)		0.0009	0.0003	2.76	0.0058
MCHC		-0.0520	0.0262	-1.99	0.0468
MCV		0. 0165	0.0050	3.27	0.0011
Coronary angiogram (yes)		-0.2332	0.1137	-2.05	0.0404
Acute renal failure (yes)		0.2276	0.0824	2.76	0.0058
Hyperlipemia (yes)		-0.3016	0.0842	-3.58	0.0003
Digoxin (yes)		0.2771	0.1272	2.18	0.0294
Kidney surgery (yes)		-0.9228	0.0835	-11.05	<0.0001

## Data Availability

The data used to support the findings of this study are included within the article.
